# Improving long-term air pollution estimates with incomplete data: A method-fusion approach

**DOI:** 10.1016/j.mex.2019.06.005

**Published:** 2019-06-12

**Authors:** Karl Chastko, Matthew Adams

**Affiliations:** Department of Geography, University of Toronto Mississauga, Ontario, Canada

**Keywords:** Log median-scaled adjustment, Temporal adjustments, Air pollution, Long-term estimates

## Abstract

Mobile air pollution monitoring is an effective means of collecting spatially and temporally diverse air pollution samples. These observations are often used to predict long-term air pollution concentrations using temporal adjustments based on the time-series of a fixed location monitor. Temporal adjustments are required because the time-series is often incomplete at each spatial location. We describe a method-fusion temporal adjustment that has been demonstrated to improve the accuracy of long-term estimates from incomplete time-series data. Our adjustment approach combines the techniques of using a log transformation to modify the air pollution samples to a near normal distribution and incorporates the long-term median of a reference monitor to mediate the effects of estimate inflation created by outliers in the data. We demonstrate the approach with hourly Nitrogen Dioxide observations from Paris, France in 2016.

Method-Fusion Benefits:

• Log transformations control for estimate inflation created by log normally distributed data.
• Adjusting data with the long-term median, rather than the mean, controls for estimate inflation.
• Produces more accurate long-term estimates than other adjustments independent of the pollutant being estimated.

## Graphical abstract

**Figure:**
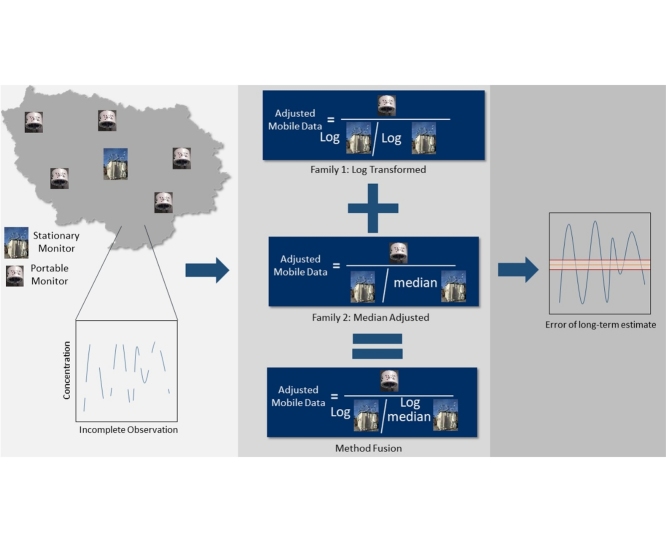

**Specifications Table**

**Table:** 

Subject Area:	*Environmental Science*
More specific subject area:	*Atmospheric Sciences, Spatial Statistics*
Method name:	*Log Median-Scaled Adjustment*
Name and reference of original method:	*Hoek, G., Meliefste, K., Cyrys, J., Lewn, M., Bellander, T., Brauer, M., Fischer, P., Gehring, U., Heinrich, J., Van Vliet, P., Brunekreef, B., 2002. Spatial variability of fine particle concentrations in three European areas. Atmos. Environ. 36, 4077–4088.* https://doi.org/10.1016/S1352-2310(02)00297-2
	*Larson, T., Henderson, S.B., Brauer, M., 2009. Mobile monitoring of particle light absorption coefficient in an urban area as a basis for land use regression. Environ. Sci. Technol. 43, 4672–4678.* https://doi.org/10.1021/es803068e
Resource availability:	*Air Parif:*https://www.airparif.asso.fr/en/

## Method details

### Introduction

Air pollution monitoring requires high-precision instruments, which are expensive and often limit the number of locations observed [1]. In addition to financial constraints, time constraints presented by monitoring multiple sites with a limited number of monitors often prevent long-term continuous monitoring, which is required to accurately estimate long-term air pollution at a given location [2]. To address these challenges, researchers often deploy portable monitors for short periods to observe air pollution at many different locations. The goal of the portable monitor use is to obtain spatially and temporally diverse data. The data collected from the short-term monitoring campaigns have been used to estimate long-term concentrations and develop predictive models of air pollution. Long-term estimates are necessary because these are the values most often used in epidemiological studies of health effects from air pollution [3,4]. Aside from calculating the raw average of short-term samples and treating these values as the long-term concentration estimate, multiplicative temporal adjustments are often employed to predict long-term exposure estimates [[5], [6], [7]].

### Multiplicative temporal adjustments

Multiplicative temporal adjustments are used to correct for temporal trends present in air pollution data collected through short term monitoring campaigns [8]. When data are collected at short intervals at different times throughout the year, or at different times throughout the day, seasonal and diurnal variation in air pollution becomes exaggerated. Multiplicative temporal adjustments account for these trends by adjusting the short-term observations against continuous data observed at a fixed-location monitoring station. For example, if data were collected during a period of above average air pollution, the adjustment would correct these observations downwards when estimating the long-term value. Applying temporal adjustments can improve accuracy of long-term pollution concentration estimates. Eq. (1) describes the basic form of a multiplicative temporal adjustment.

(1) OA=OtOFMtOCentral  Tendency  FM

Long-term estimates of air pollution are calculated by dividing each short-term observation (O*_t_*) by the ratio of its corresponding fixed monitor observation (O*_FMt_*) to the long-term central tendency of a fixed location reference monitor (O*_Central Tendency FM_*) [9].

### A method-fusion approach

**Fig. 1:**
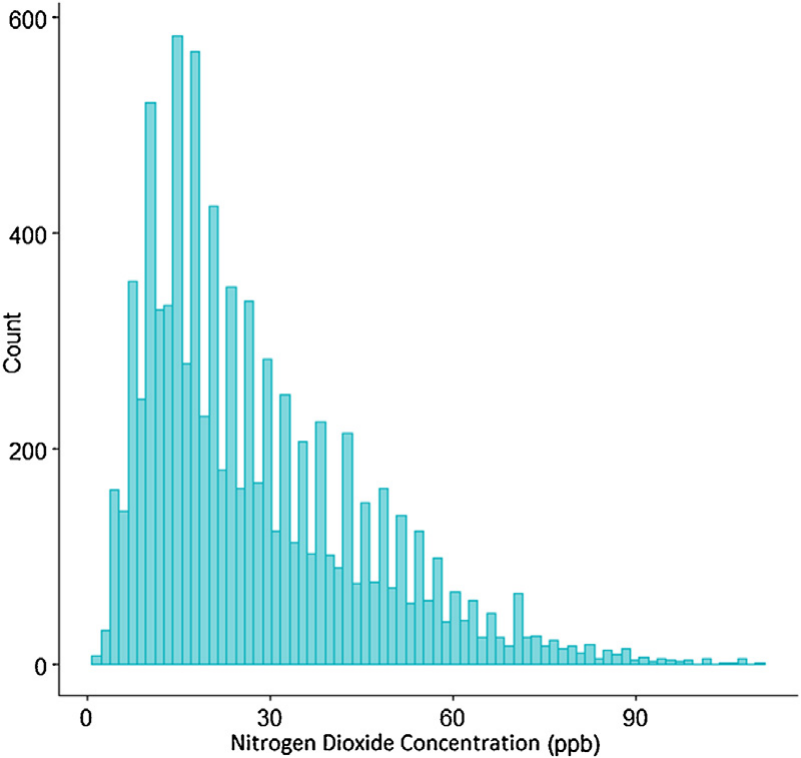
Log normal distribution of 1 year of hourly NO_2_ observations obtained from the Airparif air pollution database. Observation are from the Bobigny monitoring station in Paris France for 2016, values are reported in parts per billion.

Air pollutants are often log-normally distributed, with many low concentration observations and fewer high concentration observations, see Fig. 1.

The log-normal distribution of air pollution presents challenges when estimating long-term concentrations, due to the influence of extreme values. Two main approaches have been employed to help account for the log-normal distribution commonly present in air pollution. Approach one, see Eq. (2), utilizes the median as a measure of central tendency for the fixed location monitor [7]. By calculating the median rather than the mean, the estimates produced with this approach are less inflated by extreme values and are a better representation of the observed long-term concentration.

(2) $$O_{A}=O_{t}÷\frac{O_{FMt}}{O_{FMmedian}}$$

Approach two, see Eq. (3), applies a log transformation to the data, which transforms the data to an approximately normal distribution. This transformation minimizes the potential exaggeration of estimates produced from the presence of extreme values. This approach utilizes the mean as its measure of central tendency.

(3) OA=Otloge(OFMt+e)loge(OFMmean+e)

By incorporating elements from both approaches into one temporal adjustment, more accurate results may be obtained. Eq. (3) displays the combined temporal adjustment. Unlike other temporal adjustments, Eq. (3) utilizes both the log adjustment and median value of the fixed monitor to control for the log-normal distribution of the data being adjusted. We refer to this method-fusion approach as the log median-scaled adjustment.

(4) OA=Otloge(OFMt+e)loge(OFMmedian +e)

### Model validation

This method has been demonstrated to improve estimates compared to other multiplicative adjustments [10]. Chastko and Adams [10], simulated mobile monitoring campaigns with air pollution observations from three different cities and eight pollutants. Mobile pollution samples were adjusted using multiple temporal adjustment approaches to predict long-term concentrations for each pollutant. This analysis revealed that the log median-scaled adjustment was more accurate the all other temporal adjustments included in the study. For full details on model validation, see Chastko and Adams [10].

### Conclusion

The method fusion approach can be applied to any mobile air pollution monitoring dataset and can produce more accurate long-term estimates compared to existing temporal adjustments. These estimates are more accurate because the method fusion approach controls for inflation of the central tendency produced by log-normal distributions, which are often present in air pollution data.

### Sample workflow

The following example demonstrates how to apply the log median-scaled adjustment to a sample of air pollution data. This workflow is presented in the programming language R. For this example, mobile data will be represented as a subset of data from a stationary air pollution monitor in Paris France.

#### R libraries

To access various functions used in this demonstration, the following R libraries are loaded.

**Figure:**
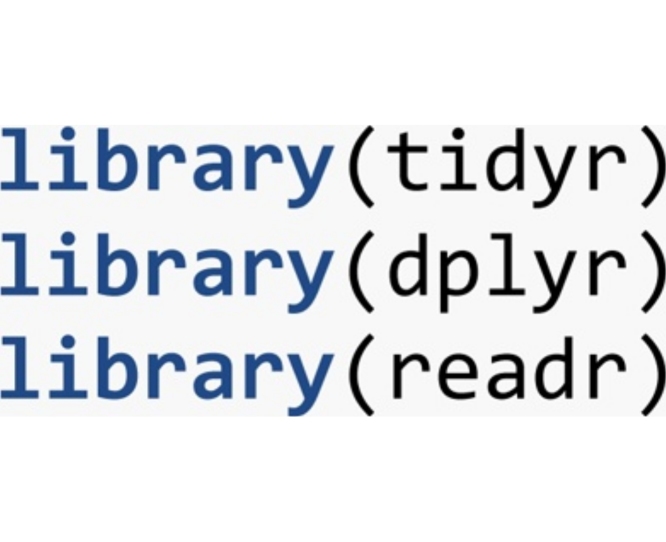

#### Loading the data

To access the sample data for this demonstration, a CSV file containing hourly Nitrogen Dioxide observations from 2016 in Paris France is loaded into R. This data is hosted on GitHub and was originally obtained from AirParif [11]. The following code block loads the data into R, assigning it to a variable **air.pollution.data**.

**Figure:**
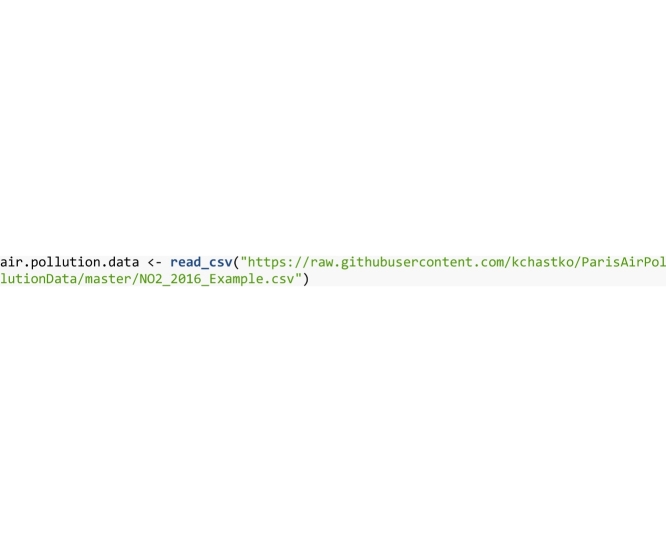

A sample of the air pollution data is provided in Table 1, it is a collection of air pollution observations from two air pollution monitors with time signatures for each observation.

**Table 1:** Sample of air pollution observation obtained from AirParif. Values represent NO_2_ concentrations measured in μg/m^3^.

Date	Hour	DateTime	Station1	Station2
1/1/2016	1	1/1/2016 0:00	35	21
1/1/2016	2	1/1/2016 1:00	42	34
1/1/2016	3	1/1/2016 2:00	55	32
1/1/2016	4	1/1/2016 3:00	54	45
1/1/2016	5	1/1/2016 4:00	50	53
1/1/2016	6	1/1/2016 5:00	47	51
1/1/2016	7	1/1/2016 6:00	47	58
1/1/2016	8	1/1/2016 7:00	46	56
1/1/2016	9	1/1/2016 8:00	40	50
1/1/2016	10	1/1/2016 9:00	31	47

#### Sampling and adjustment function

**Fig. 2:**
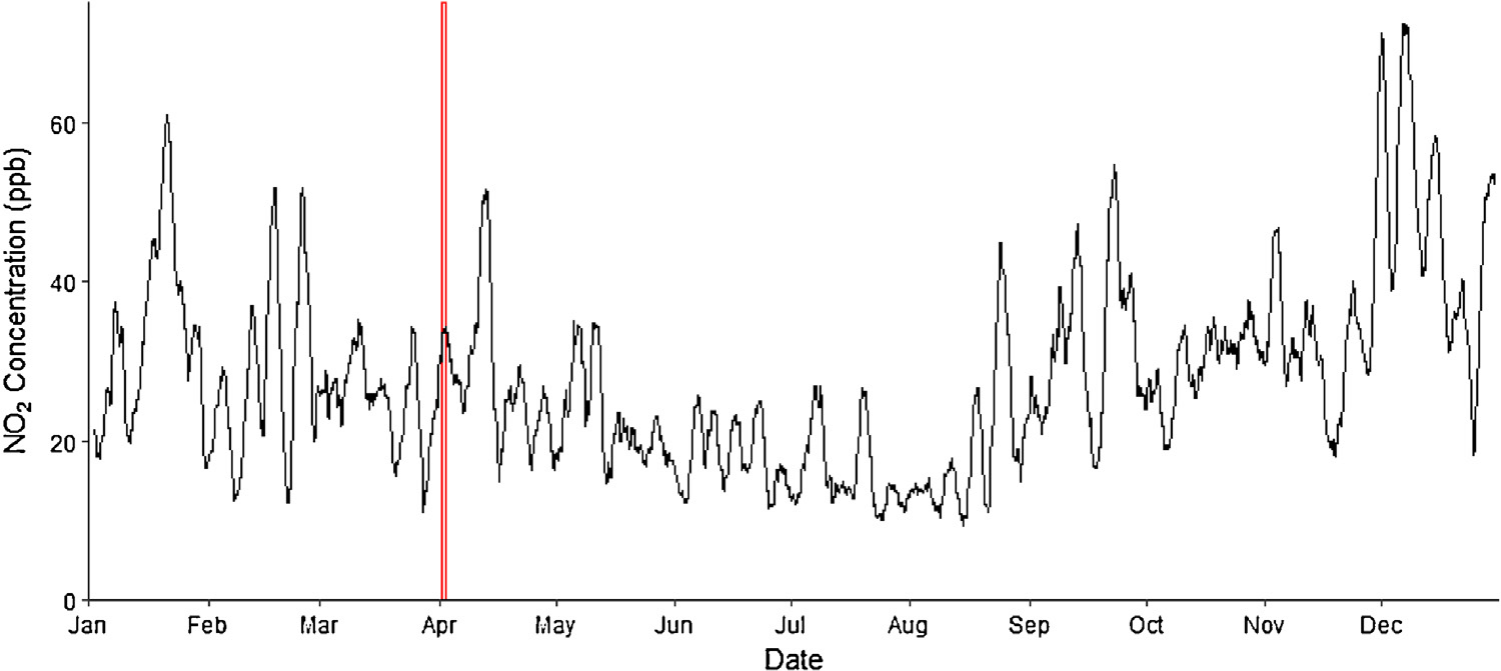
72 h moving average of NO_2_ observations in 2016 (black) and the 24 -h sample period (red) from April 1 st 2 pm to April 2nd 1 pm. All observations were recorded at the Argenteuil (Station2) air pollution monitoring station in Paris France.

With the data loaded into R, a sample of air pollution data can be taken from one of the monitors to represent the mobile data. In this example, 24 h of air pollution observations will be used. Table 2 displays the sample data and the temporally corresponding reference data that will be used to adjust each sample from the mobile data. Station 1 is used as the reference station and Station 2 is used as the sample station. Additionally, the annual median NO_2_ concentration is calculated for the reference monitor. Fig. 2 displays the time slice of mobile data in relation to the entire time series of NO_2_ values observed at Station 2.

**Table 2:** Mobile sample taken from complete time series obtained from Paris air pollution monitors.

Date	Hour	Date Time	Reference Data	Mobile Sample
1/4/2016	14	4/1/2016 14:00	19	12
1/4/2016	15	4/1/2016 15:00	20	11
1/4/2016	16	4/1/2016 16:00	27	17
1/4/2016	17	4/1/2016 17:00	25	26
1/4/2016	18	4/1/2016 18:00	30	38
1/4/2016	19	4/1/2016 19:00	40	53
1/4/2016	20	4/1/2016 20:00	60	58
1/4/2016	21	4/1/2016 21:00	62	56
1/4/2016	22	4/1/2016 22:00	61	56
1/4/2016	23	4/1/2016 23:00	40	56
1/4/2016	24	4/2/2016 0:00	46	37
2/4/2016	1	4/2/2016 1:00	58	38
2/4/2016	2	4/2/2016 2:00	63	43
2/4/2016	3	4/2/2016 3:00	60	58
2/4/2016	4	4/2/2016 4:00	60	64
2/4/2016	5	4/2/2016 5:00	62	62
2/4/2016	6	4/2/2016 6:00	60	59
2/4/2016	7	4/2/2016 7:00	50	57
2/4/2016	8	4/2/2016 8:00	62	56
2/4/2016	9	4/2/2016 9:00	48	52
2/4/2016	10	4/2/2016 10:00	30	47
2/4/2016	11	4/2/2016 11:00	22	26
2/4/2016	12	4/2/2016 12:00	15	23
2/4/2016	13	4/2/2016 13:00	16	18

**Figure:**
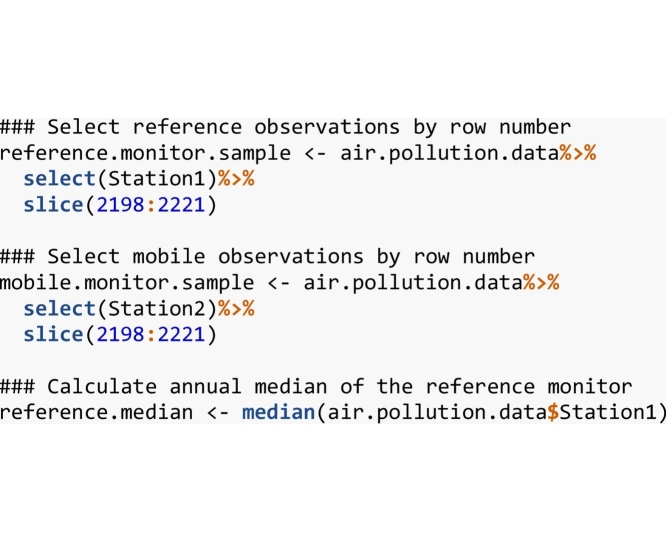

Now that all the required data have been selected, we can proceed to apply the temporal adjustment. The R implementation of the adjustment is shown below as the *LogMedianScaled* function. This function requires a vector containing the reference data, a vector containing the mobile data and the annual median pollutant concentration calculated from the entire reference dataset.

**Figure:**
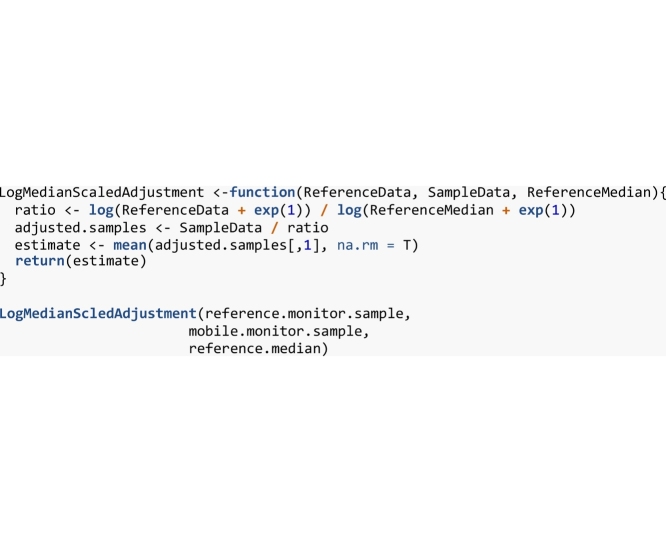

The *LogMedianScaledAdjustment* function returns a single value representing the annual average air pollution concentration estimated from the mobile sample by averaging each adjusted value in the mobile sample. Table 3 shows the adjusted values calculated by the temporal adjustment and the raw input values used to adjust the data. In this example, the raw data produce a long-term estimate of 42.62 ppb, the *LogMedianScaledAdjustment* produces an annual NO_2_ estimate of 37.15 ppb, and the actual long-term value was 30.6 ppb. By applying the log median-scaled adjustment, estimation error was reduced from 12.02 ppb to 6.55 ppb using only a 24 h sample to estimate the annual average.

**Table 3:** Temporal adjustment inputs and estimates.

Reference Data	Reference Median	Mobile Data	Adjusted Values
19	26	12	13.09
20	26	11	11.83
27	26	17	16.83
25	26	26	26.28
30	26	38	36.58
40	26	53	47.39
60	26	58	47.05
62	26	56	45.09
61	26	56	45.26
40	26	56	50.08
46	26	37	31.97
58	26	38	31.07
63	26	43	34.49
60	26	58	47.05
60	26	64	51.92
62	26	62	49.92
60	26	59	47.86
50	26	57	48.27
62	26	56	45.09
48	26	52	44.47
30	26	47	45.24
22	26	26	27.22
15	26	23	26.86
16	26	18	20.63

**Fig. 3:**
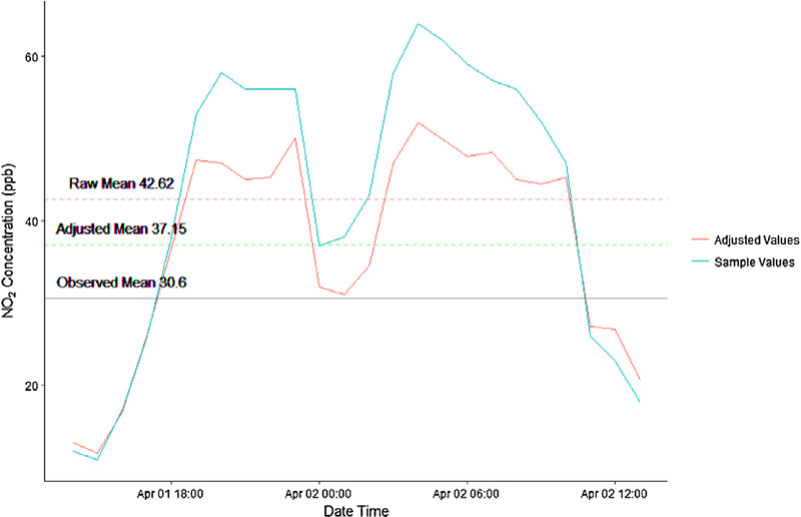
Time series of mobile sample and adjusted sample values with estimates and observed annual average. Red: Raw sample mean, Green: Adjusted mean, Black: Observed annual mean calculated from complete time series.

To visualize the accuracy of the temporally adjusted estimate we can plot the observed average annual NO_2_ value, calculated from the stationary data, alongside the raw sample average, the temporally adjusted average and the sample data. Fig. 3 shows that the temporally adjusted average is more accurate than the raw sample’s average.
